# DWV Infection *in vitro* Using Honey Bee Pupal Tissue

**DOI:** 10.3389/fmicb.2021.631889

**Published:** 2021-02-10

**Authors:** Yunfei Wu, Xuye Yuan, Jing Li, Tatsuhiko Kadowaki

**Affiliations:** Department of Biological Sciences, Xi’an Jiaotong-Liverpool University, Suzhou Dushu Lake Higher Education Town, Suzhou, China

**Keywords:** host-pathogen interaction, honey bee, deformed wing virus, viral infection, viral inhibitor

## Abstract

The deformed wing virus (DWV) has been best characterized among honey bee viruses; however, very little is known regarding the mechanisms of viral infection and replication due to the lack of immortalized honey bee cell lines. To solve this problem, we established an *in vitro* system using honey bee pupal tissue to reconstruct DWV binding and entry into the host cell, followed by translation of the RNA genome and polyprotein processing using RNA-dependent RNA polymerase (RdRP) as a marker. Using this system, the P-domain of the virion subunit VP1 was found to be essential for DWV infection, but not for binding and entry into the cell. DWV efficiently infected the head tissue derived from early but not late pupa, suggesting that undifferentiated cells are targeted for viral infection. Furthermore, we found that inhibitors of mammalian picornavirus 3C-protease, rupintrivir and quercetin suppressed RdRP synthesis, indicating that this *in vitro* system is also useful for screening a compound to control viral infection. Our *in vitro* system may help to understand the mechanism of DWV infection in host cells.

## Introduction

Large-scale loss of managed honey bee (*Apis mellifera*) colonies has been recently reported in several developed countries (Goulson et al., 2015). Honey bee pollination is important for maintaining ecosystems and the production of many crops (Klein et al., 2007; Aizen and Harder, 2009; Garibaldi et al., 2013); therefore, the prevention of the loss of honey bee colonies has become a focus in both apiculture and agriculture. Colony loss has often been associated with the ectoparasitic mites *Varroa destructor* and *Tropilaelaps mercedesae*, which feed on honey bees and transmit honey bee viruses, particularly the deformed wing virus (DWV) to the host (de Miranda and Genersch, 2010; Rosenkranz et al., 2010; Chantawannakul et al., 2018). In the absence of mites, DWV copy numbers remain low in honey bees without specific symptoms (covert infection). However, DWV levels associated with honey bees are dramatically increased in mite-infested colonies (Shen et al., 2005; Forsgren et al., 2009; Khongphinitbunjong et al., 2016; Wu et al., 2017). Honey bees often show multiple symptoms (overt infection), including the death of pupae, deformed wings, shortened abdomen, and reduced lifespan (Tentcheva et al., 2004; Yue et al., 2007; de Miranda and Genersch, 2010; Rosenkranz et al., 2010). Thus, winter colony loss is strongly correlated with the presence of DWV and *V. destructor* (Highfield et al., 2009; Nazzi and Le Conte, 2016).

DWV belongs to the *Iflaviridae* family and is a non-enveloped icosahedral virion approximately 30 nm in diameter, which contains a positive-strand RNA genome of ∼10,000 nt. The RNA genome is translated into a polyprotein that is co-translationally and post-translationally cleaved by the viral protease to produce structural and nonstructural proteins (Lanzi et al., 2006). The DWV virion is constructed from subunits VP1, VP2, and VP3, which are arranged into a capsid with pseudo-T3 icosahedral symmetry. Structural analysis of DWV virions through X-ray crystallography and cryogenic electron microscopy showed that the P-domain of VP1 (amino acid 748–901 of DWV polyprotein) is present at the outermost surface of the virion and undergoes a conformational change under different pH conditions. It may bind to the viral receptor or disrupt the membrane to deliver genomic RNA into the cytosol (Organtini et al., 2017; Skubnik et al., 2017).

Although DWV has been best characterized among honey bee viruses, very little is known regarding how the virus binds, enters, and replicates in the host cell. In fact, none of the non-structural proteins have been studied for their functions in viral replication. The DWV can propagate in honey bee larvae and pupae by viral injection (Lamp et al., 2016; Gusachenko et al., 2020; Ryabov et al., 2020); however, this *in vivo* system does not allow us to study the underlying mechanisms of viral infection and replication. Virus-free immortalized honey bee cell lines provide the best resource to study viruses and other pathogens (Guo et al., 2020); however, these cell lines are not yet available. To solve this problem, we developed an *in vitro* system to reconstitute the DWV infection using cultured honey bee pupal tissue and RNA-dependent RNA polymerase (RdRP) as a marker. We used the dissected head tissue rather than dispersed cells to make the preparation as simple as possible. RdRP is a non-structural viral protein required for replication of viral genomic RNA. RdRP is only present in the host cell where DWV infects and replicates, and thus can be used as a marker to quantify DWV-infected cells. Detection of negative-strand RNA of the DWV genome through RT-PCR has been commonly used as evidence of viral replication; however, this assay has the potential problem of false-priming artifacts (de Miranda et al., 2013). The mechanistic insight into DWV infection obtained with our *in vitro* system is reported and discussed in this study.

## Results

### Infection of Honey Bee Pupal Tissues With DWV

**FIGURE 1:**
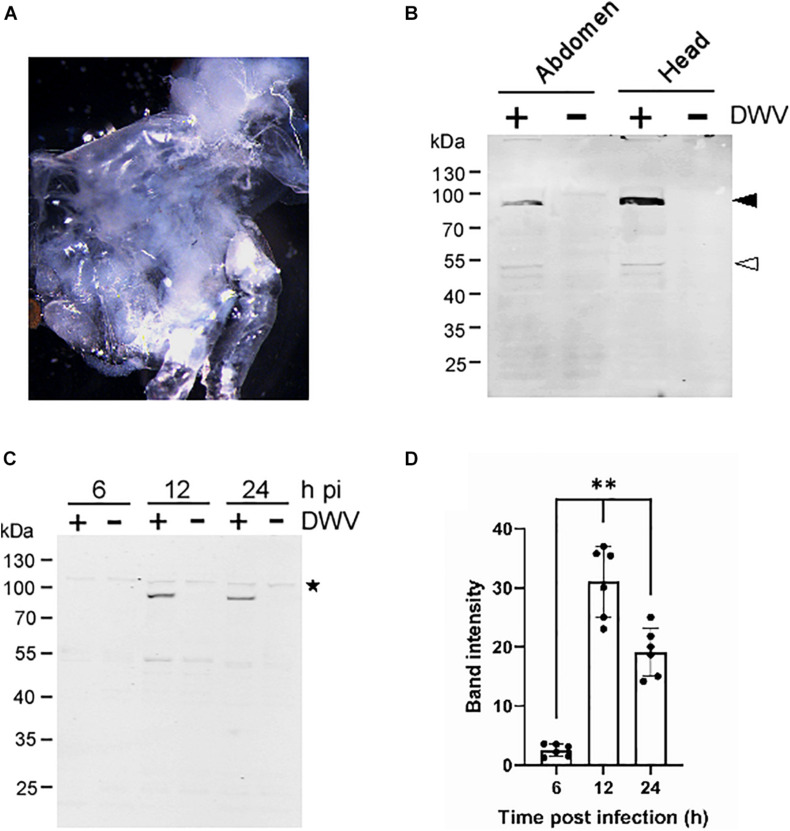
Infection of honey bee pupal tissues with DWV. **(A)** Image of the cultured head tissue from pale eyes-pupa. **(B)** Abdomen or head of the single pupa was sagittally halved and one tissue was infected by DWV (+) whereas the other was left untreated (−). DWV infection was tested through western blotting using anti-RdRP antibody. RdRP precursor with 3C-protease (90 kDa) and mature RdRP (53 kDa) bands are indicated with black and white arrowheads, respectively. The molecular weight (kDa) of the protein marker is at the left. **(C)** Synthesis of RdRP in the pupal head tissue was tested at 6, 12, and 24 h after DWV infection (hpi). The star represents a protein that non-specifically cross-reacted with anti-RdRP antibody. The presence of this band indicates that an equal amount of protein was loaded in each lane. **(D)** Band intensity of the RdRP precursor was compared between three time points using the one-tailed Dunnett test and *P*-values between 6 and 12 or 24 h are <1.3 × 10^–6^ and <7.2 × 10^–6^, respectively (**). Mean values ± SD (error bars) are shown (*n* = 6). *This represents statistical significance.

To establish a method to characterize DWV infection in honey bee cells *in vitro*, we sagittally dissected the head and abdomen of pupa with pale or pink eyes and infected the dissected tissue with concentrated DWV in the culture medium (Figure 1A). We then measured the fraction of DWV-infected cells by quantifying RdRP through western blotting. As shown in Figure 1B, two bands corresponding to the RdRP precursor with 3C-protease (3C-Pro; 90 kDa) and mature RdRP (53 kDa) were specifically detected in the head and abdominal tissues infected with DWV. The RdRP precursor was more abundant than the mature protein, suggesting that the cleavage between RdRP and 3C-Pro is rate-limiting as in other picornaviruses (Jiang et al., 2014). The RdRP precursor was not detected at 6 h but increased at 12 and 24 h after infection of pupal head tissue (Figures 1C,D). These results demonstrate that DWV infects cells in honey bee pupal tissue *in vitro*. Using RdRP as a marker, we could study the mechanism of DWV infection: the binding and entry to the host cell followed by translation of genomic RNA and polyprotein processing.

### DWV Infection of Honey Bee Pupal Head Tissue at Different Developmental Stages

**FIGURE 2:**
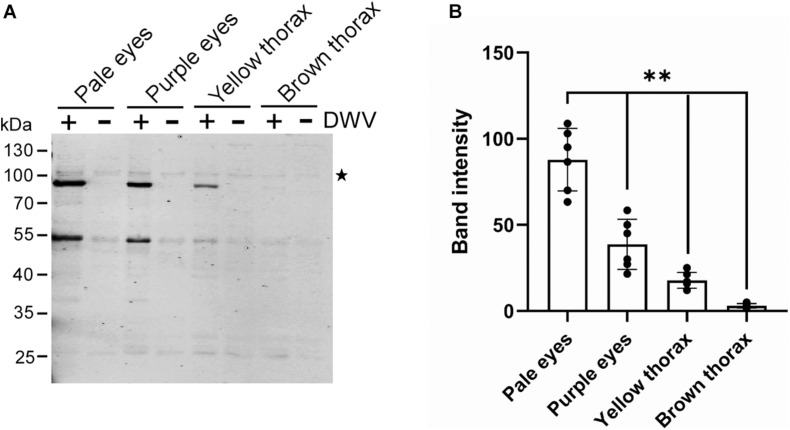
DWV infection of honey bee pupal head tissues at the different developmental stages. **(A)** Pupal head tissues at four different developmental stages (pupa with pale eyes, purple eyes, yellow thorax, and brown thorax) were infected with DWV and then RdRP synthesis was tested. **(B)** Band intensity of the RdRP precursor was compared between the four groups using the one-tailed Dunnett test and *P*-values between pale eyes and purple eyes, yellow thorax, or brown thorax are <2.2 × 10^–6^, < 1.3E × 10^–6^ < 1.3 × 10^–6^, respectively (**). Mean values ± SD (error bars) are shown (*n* = 6). *This represents statistical significance.

We tested whether the efficiency of the DWV infection changes with the developmental stage of honey bee pupal head tissue to identify the potential host factor(s) involved in DWV infection. Progression of honey bee pupal development can be monitored through the eye and thorax colors. We infected the head tissues from pupae with pale eyes (early stage), purple eyes, yellow thorax, and brown thorax (late stage) with DWV. The synthesis of the RdRP precursor decreased at later developmental stages of pupal head tissues (Figures 2A,B). Development of the pupal head was confirmed by characterizing the differentially expressed genes between pale eyes- and brown thorax-pupae. We found that 1,332 and 529 genes were up- and downregulated, respectively in the head of brown thorax-pupae (Supplementary Figures 1, 2 and Supplementary Table 1). Among the upregulated genes, gene ontology (GO) terms related to transporters, ion channels, sensory perception, and synaptic signaling were enriched. Meanwhile, GO terms associated with development, transcription, cell cycle, and microtubules were enriched in the downregulated genes (Supplementary Table 2). These results demonstrate that the cells in the head of pale eyes-pupa start developmental programs by expressing many transcriptional regulators, whereas many cells in the head of brown thorax-pupa are terminally differentiated to neurons, for example. DWV infection becomes inefficient with cells in the honey bee head tissue by the progression of pupal development.

### Role of the VP1 P-Domain in DWV Infection

**FIGURE 3:**
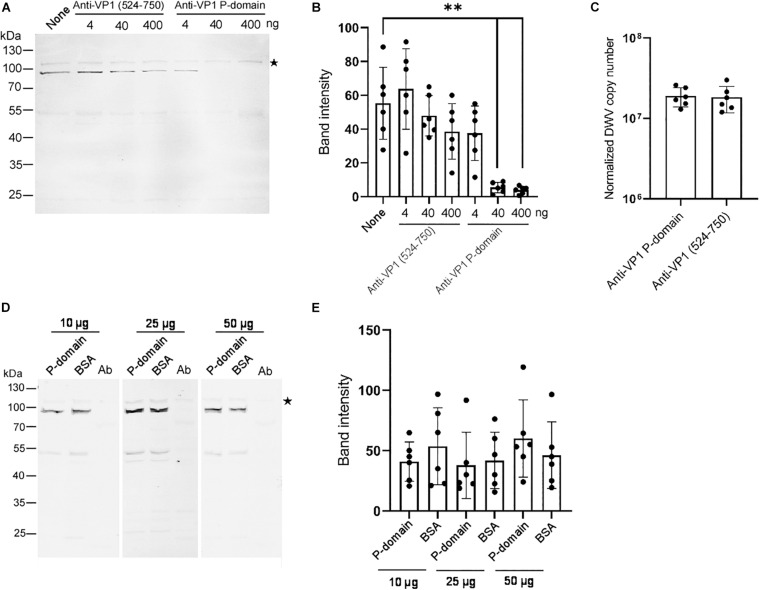
Essential role of the VP1 P-domain in DWV infection. **(A)** Effect of preincubation of DWV with 4, 40, or 400 ng of either anti-VP1 (524–750) or anti-VP1 P-domain antibody before infection on RdRP synthesis. DWV with no antibody preincubation (none) was used as a control. **(B)** Band intensity of the RdRP precursor was compared between the seven groups with the one-tailed Dunnett test. *P*-values between none and 40 or 400 ng of anti-VP1 P-domain antibody are <8.3 × 10^–6^ and <5.5 × 10^–6^, respectively (**). Mean values ± SD (error bars) are shown (*n* = 6). **(C)** Entry of DWV pre-incubated with 40 ng of either anti-VP1 (524–750) or anti-VP1 P-domain antibody to the pupal head tissues was compared (normalized copy number of DWV inside the infected cells). The difference between the two groups was not statistically significant. Mean values ± SD (error bars) are shown (*n* = 6). **(D)** Effect of preincubating pupal head tissues with 10, 25, or 50 μg of purified P-domain protein before DWV infection on RdRP synthesis. BSA was used as control. Lysate of abdomen from the same pupa (Ab) was also analyzed to confirm the lack of replication of endogenous DWV. **(E)** Band intensity of RdRP precursor was compared between P-domain and BSA with the different amount of protein. The difference between the two groups was not statistically significant. Mean values ± SD (error bars) are shown (*n* = 6). *This represents statistical significance.

To understand the role of the P-domain in DWV infection using our *in vitro* system, we pre-incubated DWV with anti-VP1 P-domain antibody and then infected the pupal head tissue. The antibody bound DWV virions under native condition because it immunoprecipitated VP1, VP2, and VP3 (Supplementary Figure 3). Another antibody recognizing the different VP1 domain (amino acids 524–750 of the DWV polyprotein) that does not bind DWV virions under native condition (Supplementary Figure 4), was used as a control. Increasing the amount of the anti-VP1 P-domain but not the anti-VP1 (524–750) antibody suppressed the synthesis of the RdRP precursor in the pupal head tissue (Figures 3A,B). These results indicate that the P-domain of VP1 plays an essential role in DWV infection.

To elucidate the mechanism of the inhibition of DWV infection by the anti-VP1 P-domain antibody, we tested the entry of the antibody-pre-incubated DWV to the cells in the pupal head tissue for 2 h. RdRP did not accumulate sufficiently during this period (Figures 1C,D). As shown in Figure 3C, viral entry into the cells was comparable between DWV pre-incubated with the anti-VP1 P-domain and anti-VP1 (524–750) antibodies, indicating that masking the P-domain with the antibody does not affect viral binding and entry. If the P-domain binds the viral receptor on the cell surface, the purified P-domain protein and DWV must compete for receptor binding. However, this was not the case as RdRP precursor synthesis was not affected by preincubating pupal head tissue with an excess amount of purified P-domain protein (Figures 3D,E). These results demonstrate that the P-domain of VP1 is essential for DWV infection but not for the binding of the viral receptor or its subsequent entry into the cell.

### Identification of Inhibitors of the DWV Infection

**FIGURE 4:**
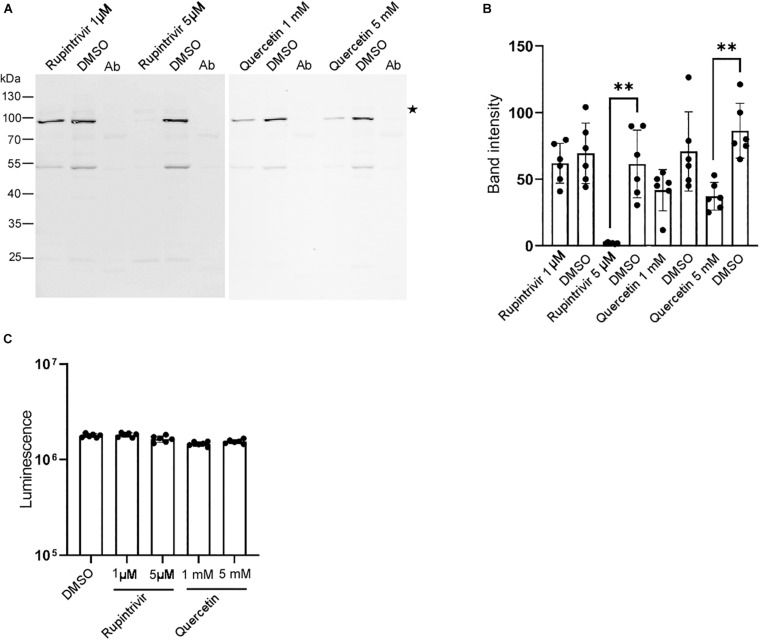
Rupintrivir and quercetin suppress RdRP synthesis in DWV-infected pupal head tissue. **(A)** Effect of rupintrivir (1 and 5 μM) and quercetin (1 and 5 mM) on RdRP synthesis in DWV-infected pupal head tissues. DMSO was used as control. **(B)** The band intensity of the RdRP precursor was compared between DMSO and rupintrivir or quercetin at different concentrations through one-tailed Welch’s *t*-test and *P*-values between DMSO and 5 μM rupintrivir or 5 mM quercetin are <0.003 and <0.002, respectively (**). Mean values ± SD (error bars) are shown (*n* = 6). **(C)** Luminescence generated by the luciferase activity dependent on the intracellular ATP level was compared between the dispersed pupal head cells treated with DMSO, rupintrivir, or quercetin at the indicated concentrations. Mean values ± SD (error bars) are shown (*n* = 6). **This represents statistical significance.

Our *in vitro* system allowed us to identify inhibitors of the DWV infection. Thus, we tested the effects of known inhibitors of mammalian picornaviruses (Baggen et al., 2018; Owino and Chu, 2019) on DWV infection. As shown in Figures 4A,B, the 3C-Pro inhibitors rupintrivir (Binford et al., 2005) and quercetin (Yao et al., 2018) decreased the synthesis of the RdRP precursor at 5 μM and 5 mM, respectively. This is consistent with the essential role of 3C-Pro in the cleavage of the viral polyprotein and host proteins (Sun et al., 2016). Neither compound dramatically affected the viability of dispersed cells derived from pupal head tissues at these concentrations (Figure 4C), suggesting that they suppressed the synthesis of the RdRP precursor by specifically inhibiting 3C-Pro.

## Discussion

The replication of DWV has been primarily characterized by the detection of the negative-strand RNA (de Miranda et al., 2013); however, the roles of non-structural proteins in the mechanism of viral infection and replication have not been studied. Using RdRP as a marker, we demonstrated that DWV infected cells in cultured honey bee pupal tissues. The cells in these tissues are unlikely to support long-term viral replication. Nevertheless, it is possible to use this *in vitro* system to study several steps of the mechanism of DWV infection, such as: (1) binding and entry of DWV into the host cell, (2) uncoating of the viral RNA genome into the cytosol, and (3) translation of the viral genomic RNA followed by polyprotein processing.

DWV infection becomes inefficient in pupal head tissue during late development. These results suggest that DWV efficiently infects and replicates in undifferentiated but not in differentiated cells. The host factors necessary for viral infection may decrease during cell differentiation. Alternatively, differentiated cells in the late pupal tissue may contain more factor(s) to repress DWV infection. This is consistent with a previous study reporting that the injection of a higher dose is required in adult bees to detect DWV at a later stage compared to that in pupae (Mockel et al., 2011). Thus, DWV efficiently infects and replicates in early pupae when introduced by parasitic mite (both *V. destructor* and *T. mercedesae*) during the reproductive phase. DWV is usually present as a covert swarm of the variants; however, mite infestation has been shown to dramatically decrease viral diversity (Martin et al., 2012). This might be due to the selective replication of the DWV variant exogenously introduced by the mite, followed by taking over the viral population in early pupae.

We showed that masking the P-domain of VP1 with the antibody inhibits DWV infection without affecting entry into the host cell. These results suggest that the P-domain does not appear to bind viral receptor on the cell surface. This is also consistent with the lack of inhibition of DWV infection by the preincubation of pupal head tissue with an excess amount of purified P-domain protein. The P-domain is likely to be necessary for the release of the DWV RNA genome into the cytosol (uncoating) after viral entry. Consistent with this hypothesis, the P-domain was suggested to contain the putative catalytic amino acids for lipase, protease, and esterase, and undergo a large conformational movement under low pH conditions (Organtini et al., 2017; Skubnik et al., 2017). DWV may bind the host cell non-specifically, followed by entry through endocytosis, and then the P-domain creates a pore in the endosomal membrane to deliver the RNA genome to the cytosol for translation.

Using our *in vitro* system, we demonstrated that 3C-Pro inhibitors of mammalian picornaviruses, rupintrivir and quercetin, reduce the synthesis of the RdRP precursor. Both compounds have been shown to directly bind 3C-Pros of rhinovirus and enterovirus (Costenaro et al., 2011; Lu et al., 2011; Wang et al., 2011; Yao et al., 2018), suggesting that DWV, rhinovirus, and enterovirus 3C-Pros share a similar structure and amino acids interacting with the compounds. Nevertheless, rupintrivir and quercetin are effective against infection of mammalian picornaviruses at much lower concentrations (in the range of nM and μM, respectively) (Binford et al., 2005; Yao et al., 2018) than DWV. Once the structure of DWV 3C-Pro becomes available, it would be possible to design inhibitors that are more potent by modifying the above compounds. We also tested the effects of other inhibitors, such as arbidol (Herod et al., 2019) and 6,7-dichloroquinoline-5,8-dione (Jung et al., 2018); however, these compounds did not affect the synthesis of the RdRP precursor. These results indicate that it is feasible to conduct large-scale screening for compounds capable of inhibiting or stimulating DWV infection using our *in vitro* system. The system might help to elucidate the mechanism of DWV infection in honey bee cells. Nevertheless, establishing immortalized honey bee cell lines (virus-free) is still useful for reproducing the complete cycle of DWV infection and replication *in vitro*.

## Materials and Methods

### Purification of DWV

46 pale eyes-pupae from a mite-free colony were injected by DWV (10^5^ copy/pupa) and incubated at 33°C for 2 days. They were homogenized with 50 mL PBS, and then the supernatant collected after centrifugation was filtered through 0.22 μm nylon membrane. The cell lysate was centrifuged through a cushion of 50% (w/v) sucrose with 36,000 rpm at 4°C for 4 h (Beckman SW41 Ti rotor). The pellet was resuspended with PBS and the copy number of DWV was quantified by qRT-PCR. DWV used for this study was one of type A strains (VD-B7) we previously isolated from *Varroa* mite-infested pupa (Wu et al., 2017). ABPV, BQCV, CBPV, IAPV, KBV, and SBV were not detected by RT-PCR.

### Quantification of DWV by qRT-PCR

DWV copy number was determined by qRT-PCR using a Hieff^TM®^ qRT-PCR SYBR Green Master Mix (Low Rox Plus, Yesen) and DWV #1 primers (Supplementary Table 3). To prepare a standard curve for DWV, PCR product obtained by above primers was purified and the copy number was determined by a formula below.

$$\mathrm{Copy}\,\mathrm{number}=\,\frac{\begin{array}{c} \mathrm{DNA}\mathrm{concentration}\left(\text{ng}/\mu l\right)\times 6.02\times \\ 10^{23}\,\,\left(\text{copies}/\text{mol}\right) \end{array}}{\begin{array}{c} \text{Length}\left(\text{bp}\right)\times 6.6\times \\ 10^{11}\,\,\left(\text{ng}/\text{mol}\right) \end{array}}$$

6.6 × 10^11^ ng/mol is the average molecular mass of one base pair and 6.02 × 10^23^ copies/mol is Avogadro’s number. We conducted qPCR using 10^1^–10^9^ copy number of the PCR product and then plotted the Ct values against the log values of copy numbers. DWV copy number in the sample was determined using the standard curve.

### Raising Anti-VP1 (524–750) Antibody

The partial VP1 cDNA corresponding to amino acid 524–750 of DWV polyprotein was amplified by PCR using two primers, 5′-SacI-VP1 and 3′-HindIII-VP1 (Supplementary Table 3). The amplified PCR product was digested by SacI (NEB) and HindIII (NEB), and then subcloned to pCold I vector (TAKARA) followed by transformation to BL21. The transformed BL21 was grown in 1 L of LB medium containing 1% glucose and 0.1 mg/mL Ampillicin at 37°C until A_600_ reached to 0.5. The cell suspension was cooled down, and then IPTG was added at 0.5 mM to induce the protein expression at 15°C for 16 h. *E. coli* was collected by centrifugation and resuspended in 100 mL of 50 mM NaH_2_PO_4_, 300 mM NaCl, 1 mM DTT, 0.1% sarkosyl, pH 8.0 containing protease inhibitors. The cell lysate was prepared by sonication using Q700 Sonicator (Qsonica) at amplitude 100 on ice for 45 min (30 s pulse with 3 min-interval). His-tag protein purification resin (Beyotime) was added to the supernatant collected after centrifugation. After gently rotating at 4°C for 2 h, the resin was washed five times with 10 mL of 50 mM NaH_2_PO_4_, 300 mM NaCl, 2 mM imidazole, 0.1% sarkosyl, pH 8.0. The bound protein was eluted with 16 mL of 50 mM NaH_2_PO_4_, 500 mM NaCl, 250 mM imidazole, 0.1% sarkosyl, pH 8.0. The eluted protein was dialyzed against 2 L of PBS with 0.1% sarkosyl, and then concentrated using Vivaspin^®^ 6 polyethersulfone 10 kDa (Sartorius). The purified and concentrated protein was delivered to GeneScript-Nanjing to raise the anti-rabbit polyclonal antibody.

### Infection of Honey Bee Pupal Tissue by DWV

Honey bee pupae with pale/pink eyes were collected from a mite-free colony. They were surface sterilized by washing with 10% bleach followed by sterile PBS three times (5 min for each wash). The head was sagittally dissected to approximately half and each tissue was suspended in 100 μL of Grace medium containing 10% FBS and antibiotics (penicillin and streptomycin) with or without DWV (9.4 × 10^8^ copy) in 24-well plate at 33°C for 1 h. Fresh culture medium (400 μL) was then added and incubated at 33°C for 16 h. DWV was pre-incubated with either anti-VP1 P-domain (Wu et al., 2020a) or anti-VP1 (524–750) antibody at the indicated amount of protein for 30 min, and then added to the pupal head tissue for infection as above. The dissected pupal head tissue was pre-incubated with either purified VP1 P-domain protein (Wu et al., 2020a) or BSA at the indicated amount of protein in Grace culture medium at room temperature for 1 h. DWV was then added for infection as above. Rupintrivir, Quercetin, or DMSO was added to the pupal head tissue at the indicated concentration together with DWV as above.

### Western Blot

After DWV infection, the pupal head tissue was collected by centrifugation and washed three times with PBS. It was then homogenized with 150 μL of RIPA buffer (20 mM Tris-HCl, pH 7.5, 150 mM NaCl, 1% NP-40, 0.5% sodium deoxycholate, 0.1% SDS) containing protease inhibitors. DWV-infected pupal abdominal tissue was homogenized with 400 μL of above buffer. After centrifugation of the homogenate, the protein concentration of supernatant was measured using Enhanced BCA protein assay kit (Beyotime). The cell lysate with 6 μg of protein was analyzed by western blot for each sample. The abdominal tissues were simultaneously tested to confirm the lack of replication of endogenous DWV in the pupa. The protein samples in SDS-PAGE sample buffer (2% SDS, 10% glycerol, 10% β-mercaptoethanol, 0.25% bromophenol blue, 50 mM Tris-HCl, pH 6.8) were heated at 95°C for 5 min. After centrifugation, the supernatants were applied to 10% SDS-PAGE and the proteins were transferred to a nitrocellulose membrane (Pall^®^ Life Sciences). The membrane was then blocked with PBST (PBS with 0.1% Tween-20) containing 5% BSA at room temperature for 1 h followed by incubating with 1,000-fold diluted anti-RdRP antibody (Wu et al., 2020a) at 4°C overnight. The membrane was washed three times with PBST (5 min each), and then incubated with 10,000-fold diluted IRDye^®^ 680RD donkey anti-rabbit IgG (H+L) (LI-COR Biosciences) in PBST containing 5% skim milk at room temperature for 1.5 h. The membrane was washed as above, and then visualized using Odyssey Imaging System (LI-COR Biosciences). Band intensity of 90 kDa RdRP precursor was measured by image-J.

### Quantification of DWV Entered to Honey Bee Cells

DWV was pre-incubated with either 40 ng of anti-VP1 P-domain or anti-VP1 (524–750) antibody for 30 min, and then added to the pupal head tissue in Grace culture medium at 33°C for 2 h. The tissue was then washed three times with PBS followed by treating with 0.5% trypsin at room temperature for 10 min to remove DWV on the cell surface. After washing three times with PBS, total RNA was extracted using TRI Reagent^®^ (Sigma-Aldrich) according to the manufacturer’s instruction. To detect DWV genome RNA, reverse transcription (RT) reaction was carried out using 1 μL of total RNA, random primer (TOYOBO), ReverTra Ace (TOYOBO), and RNase inhibitor (Beyotime). RNase H (Beyotime) was then added to digest RNA in RNA/cDNA heteroduplex after cDNA synthesis. DWV genome RNA was quantified by qRT-PCR as above. The amount of cDNA added to each qPCR reaction was normalized using *A. mellifera 18S rRNA* as the endogenous reference (Supplementary Table 3).

### Testing Cell Viability

Heads from ten pale eyes-pupae were dissected and homogenized 15 times with 10 mL Grace culture medium using Dounce homogenizer (Loose fitting). The homogenate was then filtered through a cell strainer (Falcon) and 100 μL was inoculated to each well in 96-well plate. The cells were cultured in the presence of either DMSO, Rupintrivir (1 and 5 μM), or Quercetin (1 and 5 mM) at 33°C for 16 h. The cultured plate was first incubated at room temperature for 10 min followed by adding 100 μL of reagent solution (CellTiter-Lumi^TM^ Luminescent Cell Viability Assay Kit, Beyotime). The plate was shaken at room temperature for 2 min to promote cell lysis, and then further incubated for 10 min to stabilize the chemiluminescence signal. The signal was detected using Varioskan^TM^ LUX multimode microplate reader (Thermo Fisher Scientific) and depends on intracellular ATP level, thus the relative viability of cells.

### Identification and Bioinformatic Analysis of DEGs Between the Heads of Pale Eyes- and Brown Thorax-Pupae

We sampled ten pupae with pale eyes or brown thorax from a mite-free colony. The heads of five pupae were pooled and the total RNA was extracted from each pooled sample. Thus, two samples each for pale eyes- or brown thorax-pupae were analyzed by RNA-seq. All samples were sequenced by BGISEQ-500 platform at Beijing Genomics Institute and at least 6 GB of clean data were obtained from each sample. Reference genome of honey bee, Amel_HAv3.1^1^, annotation GFF, and protein FASTA files were downloaded from NCBI. We used a gffread program^2^ to convert the honey bee GFF to GTF file, and then generated the genomic indices from the converted GTF file using genomeGenerate of STAR (Version: 2.7.0) (Dobin et al., 2013). Quality of all RNA-seq data was analyzed using FastQC (Brown et al., 2017) and pruned by Trimmomatic-0.35 (Bolger et al., 2014). After trimming the low-quality reads and removing the adapters, more than 95% of the reads in each sample were retained for the analyses. The clean reads of RNA-seq were then mapped to the STAR index. The assembled reads were mapped to honey bee genome using alignReads of STAR and the coordinated-sorted BAM format files were generated. The mapping rates of four samples to honey bee genome were between 94.12 and 94.67%. We conducted the principal component analysis of four RNA-seq samples by gmodels package (version 2.18.1) of R. We used htseq-count (Anders et al., 2015) developed with HTSeq to count the overlap of reads mapped to the GFF features for preprocess of the differential gene expression analysis. We analyzed the raw read counts in RStudio (Version 1.2.1335) using the liner contrast in DESeq2 package (Version 1.24.0) from Bioconductor (Robinson et al., 2010). Normalization was performed by the shrinkage estimation in DESeq2 (Love et al., 2014). Any reads with less than one count per million mapped reads were removed from the analysis. Benjamin-Hochberg method was applied to control false discovery rate (FDR) and log2 fold change (FC) across the detected loci. DEGs between the heads of pale eyes- and brown thorax-pupae were identified by threshold of FDR < 0.05 and log2 FC > 1. The heatmap of DEGs was generated using pheatmap package (version 1.0.12) of R. We performed the GO enrichment analysis of DEGs using clusterProfiler package (version 3.18.0) (Yu et al., 2012) and all statistical analyses were conducted by Rstudio (Version 1.2.1335). After obtaining the enriched GO terms, we identified the most specific ones by cut-off value of FDR < 0.05. Accession numbers for the RNA-seq data are SAMN16860738, SAMN16860739, SAMN16860740, SAMN16860741.

### Mass Spectrometry Analysis of Immunoprecipitates by Anti-VP1 P-Domain Antibody

Anti-VP1 P-domain antibody (75.4 μg) or pre-immune serum was first bound to Protein A-agarose (Beyotime), and then washed three times with 0.2 M sodium borate (pH 9.0). The cross-linking was conducted by mixing with 20 mM Dimethylpimelimidate (Thermo Fisher Scientific) at room temperature for 40 min with agitation. To quench the reaction, 0.2 M ethanolamine (pH 8.0) was added and agitated for 1 h. The cross-linked beads were washed three times with 150 mM NaCl containing 0.58% acetic acid followed by three washes with ice-cold PBS. The abdominal lysates of six pale eyes-pupae injected by DWV were prepared using 2 mL of RIPA buffer containing protease inhibitors. The beads were incubated with the above cell lysates at 4°C overnight, and then washed three times with buffer (150 mM NaCl, 10 mM EGTA, 0.2% NP-40, 50 mM Tris-HCl, pH 8.0) followed by additional three washes with 50 mM ammonium bicarbonate. The protein-bound beads in 50 μL of 50 mM ammonium bicarbonate were heated at 95°C for 5 min. The immunoprecipitates were applied to 8% SDS-PAGE and the gel was stained by ProteoSilver^TM^ stain kit (Sigma-Aldrich) according to manufacturer’s instruction. Four major bands specifically immunoprecipitated by anti-VP1 P-domain antibody were cut into small pieces and sequentially destained by 100 mM ammonium bicarbonate, 100 mM ammonium bicarbonate/acetonitrile (1:1 mixture), and 100% acetonitrile. The gel pieces were then reduced by 10 mM DTT in 100 mM ammonium bicarbonate solution at 56°C for 1 h and alkylated by 55 mM iodoacetamide in 100 mM ammonium bicarbonate solution for 45 min at room temperature under dark. They were dried and then swelled in 25 mM ammonium bicarbonate containing 20 ng trypsin on ice followed by overnight incubation at 37°C. Mass spectrometric detection was performed using Easy-nLC1000 coupled to LTQ Orbitrap Elite mass spectrometer (Thermo Fisher Scientific). It was equipped with nanoelectrospray source and operated in data-dependent acquisition (DDA) mode with the following settings: spray voltage 2,300 V, s-lens RF level 60%, capillary temperature 220°C, scans 350–1,800 m/z. Peptides were separated by 15 cm analytical RSLC column (Acclaim^TM^ PepMap^TM^ 100 C18 2 μm pore size, 150 mm length, 50 μm i.d.) with the gradient of 0–95% acetonitrile with 0.1% formic acid): 0–5% for 5 min, 5–25% for 40 min, 25–40% for 10 min, 40–95% for 2.5 min, and then at 95% for another 2.5 min. The ten most intense ions from full scan were selected for tandem mass spectrometry. The normalized collision energy was 35 V and default charge state was 2 in HCD mode. Scans were collected in a positive polarity mode. Tandem mass spectra were extracted by Mascot Distiller 2.7 (v2.4.1) and all MS/MS samples were analyzed using Mascot (Matrix Science, London, United Kingdom; version 2.4.1). Mascot was set up to search against *A. mellifera* and DWV protein databases with trypsin. Mascot was searched with a fragment ion mass tolerance of 0.050 Da and a parent ion tolerance of 10.0 PPM. Carbamidomethyl of cysteine was specified as a fixed modification and oxidation of methionine was specified as a variable modification. Scaffold (version Scaffold_4.9.0, Proteome Software Inc., Portland, OR) was used to validate MS/MS based peptide and protein identification. Peptide identification was accepted if FDR was <0.01 by the Scaffold Local FDR algorithm. Protein identification was accepted if FDR was <0.01 and it contained at least two identified peptides. Protein probabilities were assigned by the Protein Prophet algorithm (Nesvizhskii et al., 2003). Proteins containing similar peptides that can not be distinguished based on MS/MS analysis alone were grouped to satisfy the principles of parsimony. Proteins sharing significant peptide evidence were grouped into the clusters.

### Immunoprecipitation of Native and Denatured VP1 by Anti-VP1 (524–750) Antibody

Frozen pale eyes-pupa injected by DWV was sagittally dissected to the half and each tissue was homogenized with either 1.5 mL of RIPA buffer containing protease inhibitors (native) or 0.2 mL of denaturing buffer (1% SDS, 5 mM EDTA, 10 mM DTT) containing protease inhibitors. To prepare denatured VP1, the supernatant of lysate prepared with the denaturing buffer was heated at 95°C for 5 min followed by adding 1.8 mL of RIPA buffer containing protease inhibitors. VP1 was immunoprecipitated using anti-VP1 (524–750) antibody and Protein A-agarose at 4°C for 2 h, and then the protein-bound beads were washed five times with RIPA buffer. The beads were suspended with 100 μL of SDS-PAGE sample buffer, and then heated at 95°C for 5 min. The different volumes of the immunoprecipitates (5, 10, 15, and 20 μL) were analyzed by western blot using above antibody. Since the antibody was not cross-linked to Protein A-agarose, it was eluted from the beads together with VP1.

### Statistical Analysis

All data presented were from representative independent experiments. Statistical analyses were performed with Bell Curve for Excel (Social Survey Research Information Co., Ltd.) and no data point was excluded. The applied statistical tests and *P*-values are described in figure legends.

## Data Availability Statement

The datasets generated for this study can be found in the online repositories. The names of the repository/repositories and accession number(s) can be found in the article/Supplementary Material.

## Author Contributions

TK conceived and designed the research strategy. YW, XY, and JL performed the experiments. YW and TK analyzed the data and wrote the manuscript. All authors contributed to the article and approved the submitted version.

## Conflict of Interest

The authors declare that the research was conducted in the absence of any commercial or financial relationships that could be construed as a potential conflict of interest.

## Footnotes

1 https://www.ncbi.nlm.nih.gov/genome/?term~=~txid7460[orgn]

2 http://ccb.jhu.edu/software/stringtie/gff.shtml

## Supplementary Material

Supplementary Figure 1 Principal component analysis of four RNA-seq samples. Two RNA-seq samples each for pale eyes- (red circle) and brown thorax- (blue circle) pupae were analyzed by principal component analysis. Two samples cluster together in each group.

Supplementary Figure 2 Heatmap of the differentially expressed genes between the heads of pale eyes- and brown thorax-pupae. Expression profiles of 1,861 differentially expressed genes between the heads of pale eyes (#1 and #2)- and brown thorax (#1 and #2)-pupae are shown by the heatmap. The genes are clustered based on the expression profile indicated by the different colors (blue to red).

Supplementary Figure 3 Silver staining of immunoprecipitates by anti-VP1 P-domain antibody. Lysates of DWV-infected pupae were immunoprecipitated by either anti-VP1 P-domain antibody or the pre-immune serum. The eluted immunoprecipitates were separated by 8% SDS-PAGE and then processed for silver staining. Four major bands specifically immunoprecipitated by anti-VP1 P-domain antibody were identified to be VP1, VP2, and VP3 by the mass spectrometry analysis. VP2 and VP3 present in DWV virion were co-immunoprecipitated with VP1. The size (kDa) of protein molecular weight marker is at the right.

Supplementary Figure 4 Immunoprecipitation of native and denatured VP1 by anti-VP1 (524-750) antibody. Native and heat-denatured lysates prepared with DWV-infected pupa were immunoprecipitated by anti-VP1 (524-750) antibody. The eluted immunoprecipitates with different volume (5, 10, 15, and 20 μL) were analyzed by western blot using the same antibody. White and black arrow heads represent IgG heavy chain and VP1, respectively. VP1 is only immunoprecipitated under denatured condition. The size (kDa) of protein molecular weight marker is at the left.
