# Supplementary figures and images for: DWV Infection in vitro Using Honey Bee Pupal Tissue
Source: Front Microbiol. 2021 Feb 10;12:631889. doi: 10.3389/fmicb.2021.631889 (PMC7902917; doi:10.3389/fmicb.2021.631889)

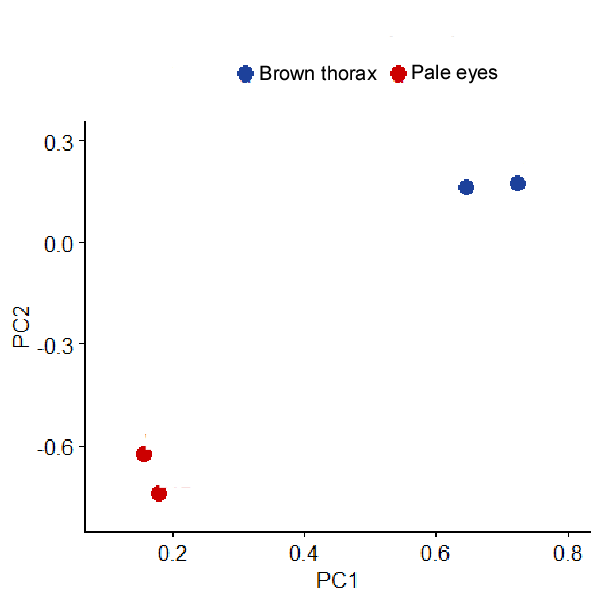

Supplement: Supplementary Figure 1 — Principal component analysis of four RNA-seq samples. Two RNA-seq samples each for pale eyes- (red circle) and brown thorax- (blue circle) pupae were analyzed by principal component analysis. Two samples cluster together in each group. [file Image_1.TIF]

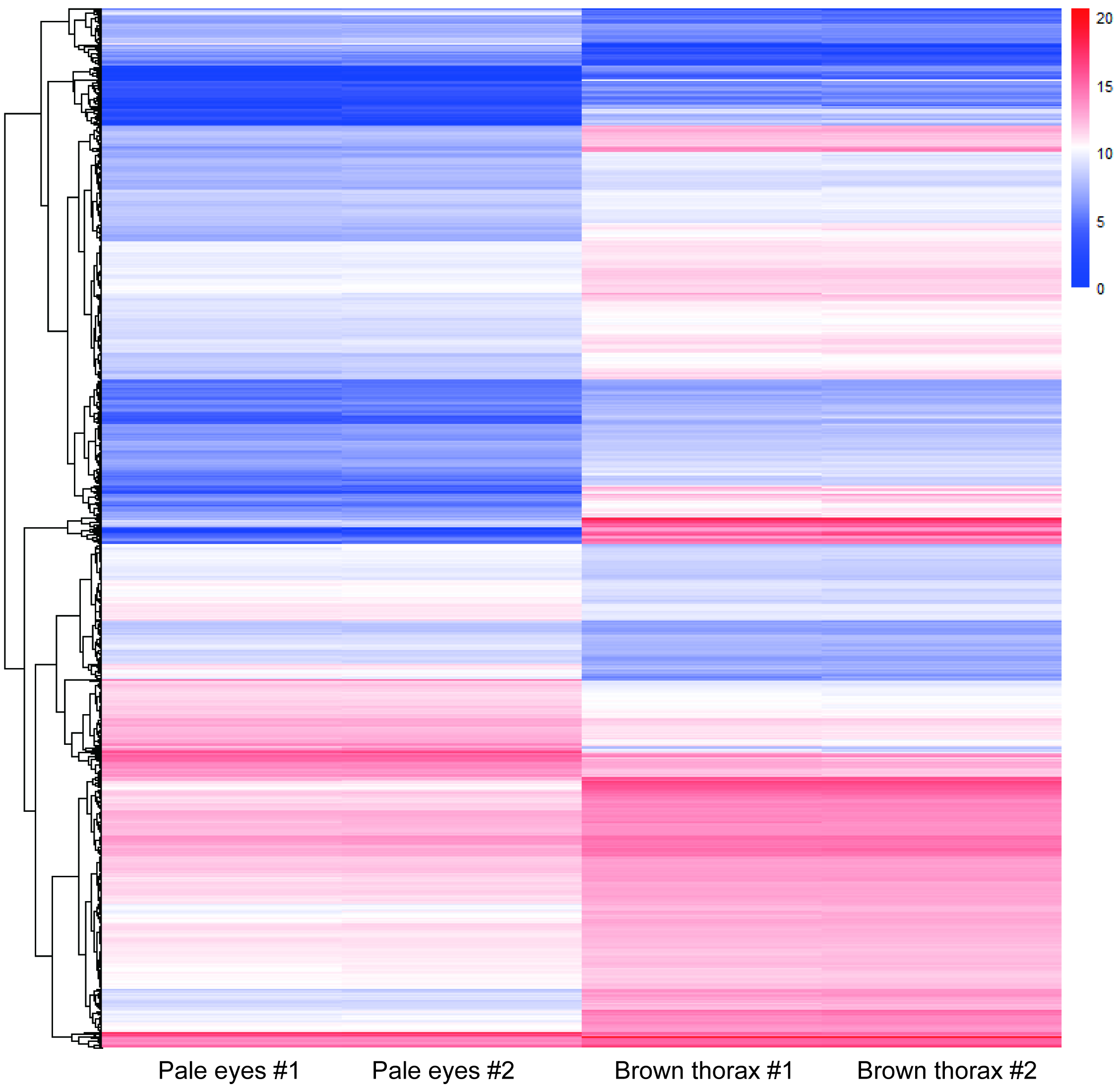

Supplement: Supplementary Figure 2 — Heatmap of the differentially expressed genes between the heads of pale eyes- and brown thorax-pupae. Expression profiles of 1,861 differentially expressed genes between the heads of pale eyes (#1 and #2)- and brown thorax (#1 and #2)-pupae are shown by the heatmap. The genes are clustered based on the expression profile indicated by the different colors (blue to red). [file Image_2.TIF]

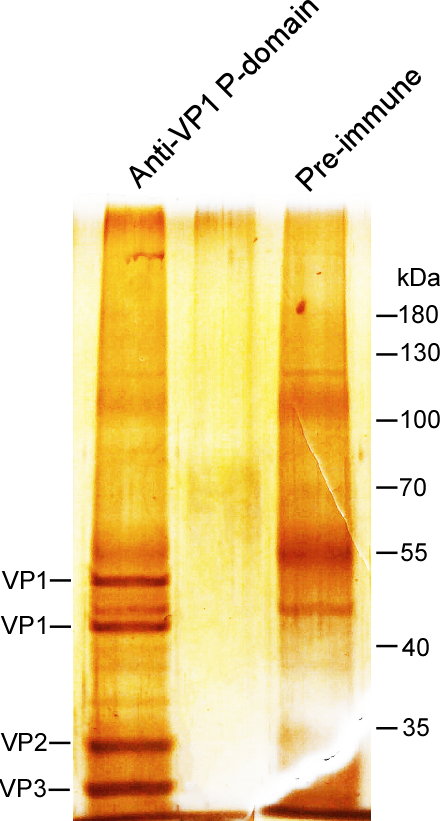

Supplement: Supplementary Figure 3 — Silver staining of immunoprecipitates by anti-VP1 P-domain antibody. Lysates of DWV-infected pupae were immunoprecipitated by either anti-VP1 P-domain antibody or the pre-immune serum. The eluted immunoprecipitates were separated by 8% SDS-PAGE and then processed for silver staining. Four major bands specifically immunoprecipitated by anti-VP1 P-domain antibody were identified to be VP1, VP2, and VP3 by the mass spectrometry analysis. VP2 and VP3 present in DWV virion were co-immunoprecipitated with VP1. The size (kDa) of protein molecular weight marker is at the right. [file Image_3.TIF]

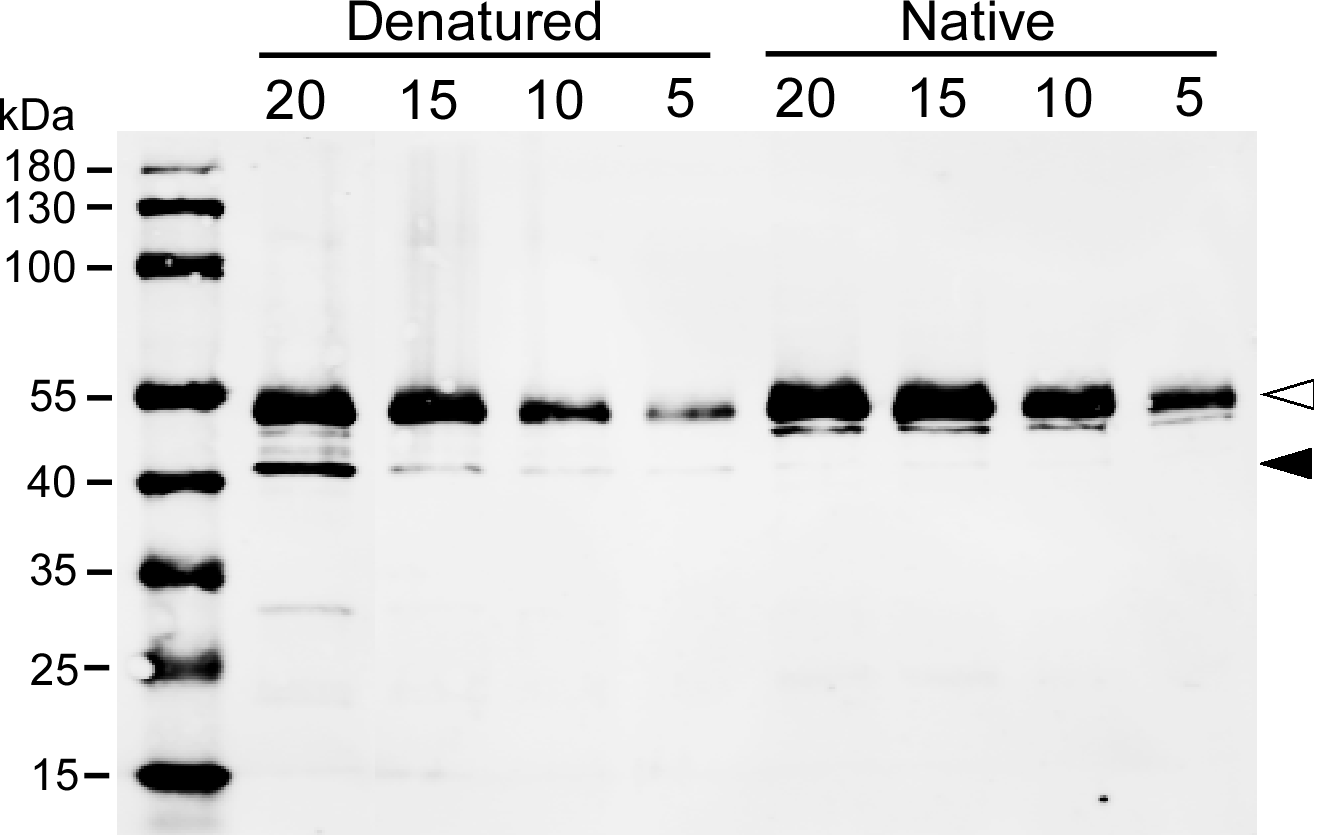

Supplement: Supplementary Figure 4 — Immunoprecipitation of native and denatured VP1 by anti-VP1 (524-750) antibody. Native and heat-denatured lysates prepared with DWV-infected pupa were immunoprecipitated by anti-VP1 (524-750) antibody. The eluted immunoprecipitates with different volume (5, 10, 15, and 20 μL) were analyzed by western blot using the same antibody. White and black arrow heads represent IgG heavy chain and VP1, respectively. VP1 is only immunoprecipitated under denatured condition. The size (kDa) of protein molecular weight marker is at the left. [file Image_4.TIF]
