# Supplementary material for: DWV Infection in vitro Using Honey Bee Pupal Tissue
Source: Front Microbiol. 2021 Feb 10;12:631889. doi: 10.3389/fmicb.2021.631889 (PMC7902917; doi:10.3389/fmicb.2021.631889)
Supplement: Supplementary file 7 [file Table_3.DOCX]

Supplementary Table 3 List of primers used in this study

| Primer | Forward (F) and reverse (R) primer sequences (5’-3’) | Product size (bp) | References |
| --- | --- | --- | --- |
| DWV #1 | (F) TTCATTAAAGCCACCTGGAACATC | 136 | Locke et al., 2012 |
|  | (R) TTTCCTCATTAACTGTGTCGTTGA |  |  |
| *A. mellifera 18S rRNA* | (F) ACCACATCCAAGGAAGGCAG | 112 | Wu et al., 2017 |
|  | (R) ACTCATTCCGATTACGGGGC |  |  |
| 5’-SacI-VP1 | TTTGAGCTCGGTACGACACAACACCCTGTAGGT | 699 | This study |
| 3’-HindⅢ-VP1 | TTTAAGCTTCTTAGCCCTGTATTCTTCGTCATT |  |  |

**Locke, B., Forsgren, E., Fries, I. and de Miranda, J. R.** (2012). Acaricide treatment affects viral dynamics in Varroa destructor-infested honey bee colonies via both host physiology and mite control. *Appl Environ Microbiol* **78**, 227-235.

**Wu, Y., Dong, X. and Kadowaki, T.** (2017). Characterization of the Copy Number and Variants of Deformed Wing Virus (DWV) in the Pairs of Honey Bee Pupa and Infesting. *Front Microbiol* **8**, 1558.
